# Success Predictors of Endoscopic Sleeve Gastroplasty

**DOI:** 10.1007/s11695-024-07109-4

**Published:** 2024-03-07

**Authors:** Maria Valeria Matteo, Vincenzo Bove, Gabriele Ciasca, Giorgio Carlino, Riccardo Di Santo, Laila Vinti, Giulia Polidori, Valerio Pontecorvi, Massimiliano Papi, Cristiano Spada, Ivo Boškoski

**Affiliations:** 1 Digestive Endoscopy Unit, Fondazione Policlinico Universitario Agostino Gemelli IRCCS, Largo A. Gemelli, 8, 00168 Rome, Italy; 2 Università Cattolica del Sacro Cuore, 00168 Roma, Italy; 3 Fondazione Policlinico Universitario A. Gemelli IRCCS, 00168 Roma, Italy; 4 Dipartimento di Neuroscienze, Sezione di Fisica, Università Cattolica Del Sacro Cuore, 00168 Roma, Italy; 5 Department of Translational and Precision Medicine, Sapienza University of Rome, Roma, Italy

**Keywords:** Obesity, Endoscopic sleeve gastroplasty, ESG, Predictors

## Abstract

**Objective:**

Endoscopic sleeve gastroplasty (ESG) is a minimally invasive procedure that proved to be safe and effective in obesity treatment. However, not all subjects respond to treatment in the same way, and, with a view to personalized care, it is essential to identify predictors of success or failure.

**Methods:**

A retrospective 2-year followed-up cohort of ESG subjects was analyzed to investigate the presence of any baseline or early indicators of long-term optimal or suboptimal ESG outcomes.

**Results:**

A total of 315 subjects (73% women) were included, with 73% of patients exhibiting an Excess weight loss percentage (%EWL) >25% at the 24 months. Neither demographic parameters (age and sex), smoking habits, and menopause in women nor the presence of comorbidities proved potential predictive value. Interestingly, the %EWL at 1 month after ESG was the strongest predictor of 24-month therapeutic success. Subsequently, we estimated an “early threshold for success” for 1 month-%EWL by employing Youden’s index method.

**Conclusions:**

ESG is a safe and effective bariatric treatment that can be offered to a wide range of subjects. Early weight loss seems to impact long-term ESG results significantly and may allow proper early post-operative care optimization.

**Graphical Abstract:**

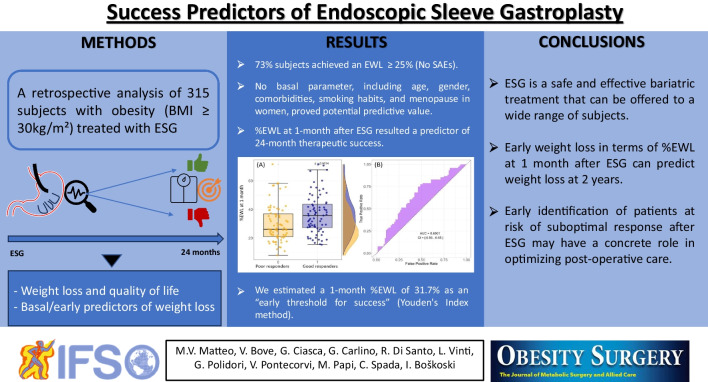

## Introduction

Obesity is a chronic relapsing disease that has reached the dimension of a pandemic. The WHO recently estimated that obesity prevalence reached 13% of the world's adult population in 2016 [1]. Obesity is associated with multiple comorbidities, including type 2 diabetes, cardiovascular diseases, and several tumors [2].

Current therapeutic options for obesity include non-invasive approaches (diet, lifestyle interventions, anti-obesity medications), endoscopy, and bariatric surgery. While medical non-invasive approaches may often fail to induce adequate and/or sustained weight loss, bariatric surgery, which is currently the most effective and durable treatment for obesity, is limited by elevated costs and non-negligible morbidity and mortality, with only 1% of suitable patients undergoing surgery [3, 4]. Endoscopic bariatric treatments have recently emerged as minimally invasive procedural options to fill the huge gap between medical and surgical treatments [5].

Endoscopic sleeve gastroplasty (ESG) is an organ-sparing bariatric procedure consisting of endoluminal full-thickness suturing of the gastric body, leading to volume restriction along with delay of gastric emptying [6, 7]. ESG proved to be safe and effective in inducing weight loss, with a mean %TBWL of about 17-20% at 2 years and 16% at 5 years, along with improvement of obesity-related comorbidities [8–13]. A recent randomized controlled trial confirmed the superiority of ESG combined with lifestyle modifications above lifestyle modifications alone, showing significant weight loss and improvement in metabolic comorbidities in the ESG group compared to controls [14]. However, not all patients achieve the same results. Adherence to follow-up, younger age, and weight loss at 1 and 6 months have been reported as predictors of weight loss in few studies [10, 13, 15]. However, since ESG is a relatively new intervention, the factors associated with better outcomes remain insufficiently explored. Identifying predictors of success or failure may improve weight loss outcomes by a smart selection of patients who are most likely to respond to ESG. Furthermore, this approach could enable the early detection of poor responders who might benefit from additional treatment. This study aims to further evaluate the trends in weight loss and quality of life following ESG, and to investigate the presence of any baseline or early indicators of long-term favorable or unfavorable responses to ESG.

## Methods

### Study Design, Ethics, and Participants

We performed a retrospective analysis of a prospective database including data on all ESG procedures performed from May 2017 to March 2022 at the Digestive Endoscopy Unit of Fondazione Policlinico Universitario A. Gemelli IRCCS in Rome. Inclusion criteria were class I obesity (boby mass index (BMI) 30–34.9 kg/m^2^) with obesity-related comorbidities, class II obesity (BMI 35–39.9 kg/m^2^) with or without obesity-related comorbidities, and class III obesity (BMI >40 kg/m^2^) refusing or unfit for bariatric surgery. Exclusion criteria included previous bariatric surgery or any other type of surgery of the esophagus, stomach, and duodenum, upper gastrointestinal organic or motility disorders (i.e., active ulcers, malformations, severe gastritis), bleeding disorders, breastfeeding, pregnancy, enrollment in other studies, active drug or alcohol abuse, active eating disorders, and other uncontrolled psychiatric disorders.

Before ESG, all patients were evaluated and identified as eligible for the bariatric procedure by the local bariatric multidisciplinary team. The institutional ethics committee approved this study (number 2083/2018). Informed consent was obtained from every patient. The study was conducted in accordance with the ethical standards of the 1964 Declaration of Helsinki and its later amendments or comparable ethical standards.

### Procedures and Data Collection

**Fig. 1:**
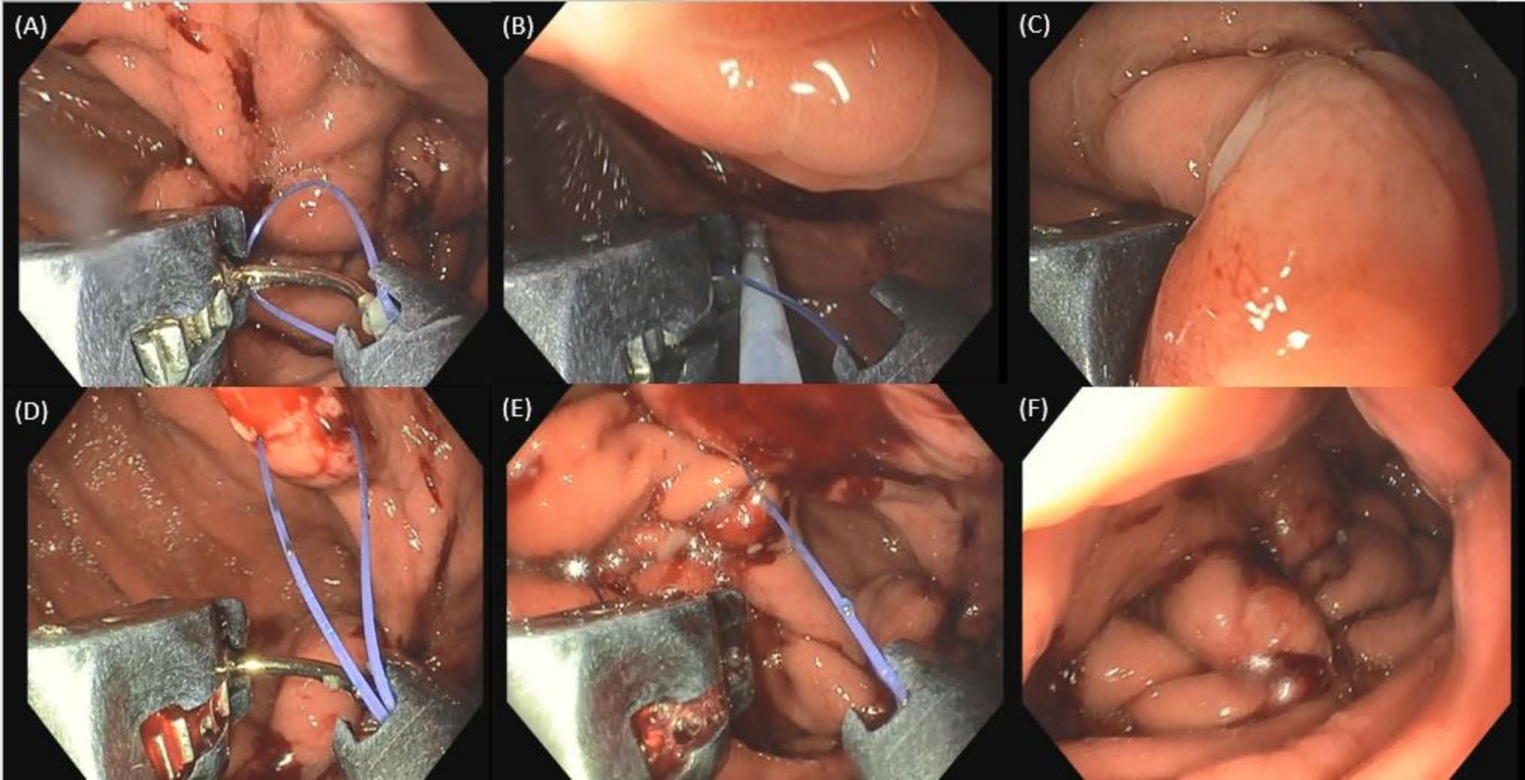
ESG performed with the OverStitch Sx mounted on a single-channel gastroscope. **A** Tip of the device (needle driver) closed to allow loading of the suture delivered from the a dedicated accessory (anchor exchange, not visible); **B** tip of the device open while grasping the gastric wall by a dedicated accessory (Tissue Helix); **C**, **D** full-thickness passage of the suture line at the anterior wall; **E** multiple full-thickness passages of the sutures along the greater curvature with a *U*-shaped pattern, before tightening with a dedicated accessory (Suture Cinch, not visible); **F** final result of ESG

All ESG procedures were conducted under general anesthesia and with CO2 inflation. Full-thickness suturing of the gastric body was performed with a *U*-shaped suture pattern by the Apollo OverStitch® (Apollo Endosurgery, Austin, TX, USA) and a double channel gastroscope (Olympus 2TGIF-160 or 2TGIF-180) or the Apollo OverStitch Sx® and a single channel gastroscope (GIF-H190) (Fig. 1). All patients were included in a multidisciplinary follow-up scheduled at 1, 3, 6, and then every 6 months, as per routine clinical practice. Subjects who did not attend two or more visits consecutively were considered “lost to follow-up.”

Demographic data, smoking habits, menopausal state for female patients, weight, body mass index (BMI), and the presence of comorbidities were collected. As the prevalence of obesity varies according to gender, partially due to biological and behavioral factors, the data were split accordingly to explore its potential predictive value and to find any differences between male and female subjects [16]. Percentage of excess weight loss (%EWL), percentage of total body weight loss (%TBWL), and quality of life changes (BAROS score [17]) were assessed during follow-up. Weight loss indices were calculated as recommended by the American Society for Metabolic and Bariatric Surgery (ASMBS) [18].

### Statistical Analysis

Data visualization and analysis were carried out using Origin Pro (version 2022) and the R software (version 4.3.1). Temporal trends of %EWL, %TBWL, and BAROS were investigated using the following double exponential model: $y={y}_0+{A}_d+{A}_g\left({e}^{-{x}_c/{t}_g}-{e}^{-x/{t}_g}\right)\ for\ x\le {x}_c,y={y}_0+{A}_d{e}^{-\left(x-{x}_c\right)/{t}_d}\ for\ x>{x}_c\ \left(\textrm{eq}.1\right)$. To explore correlations among variables, Spearman's correlation coefficient was applied, and the resulting correlation matrix was generated using the R package “corrplot” [19], in accordance with methodologies from prior research [20]. Categorical data were presented as absolute values and percentages, while quantitative variables underwent an initial assessment of data distribution through the Shapiro-Wilk normality test. Given the observed significant deviations from normality in various variables, continuous data were summarized as medians and interquartile ranges (IQR). Group comparisons for continuous variables were conducted using the Wilcoxon rank sum test, whereas percentages were subjected to analysis using the Fisher’s exact test or Pearson’s Chi-squared test, as deemed appropriate.

## Results

In total, 315 patients (median age 46 years) selected by the local bariatric multidisciplinary team underwent ESG between May 2017 and March 2022. No severe procedure-related adverse events were recorded. The follow-up rate was 97.4% (307/315) at 1 month, 87.9% (277/315) at 6 months, 84.7% (267/315) at 12 months, and 58.1% (183/315) at 18-24 months. To note, approximately 30% of patients were still within the process of reaching the 2-year follow-up and should not be classified as “lost to follow-up.”

Patients’ characteristics at baseline are summarized in Table 1, stratified by females (*N* = 230) and males (*n* = 85). An analysis of Table 1 shows that within our 24-month-followed cohort, males are significantly older than females. The two cohorts did not show statistically significant differences in the prevalence of diabetes and hyperinsulinemia. On the other hand, males display a significantly higher prevalence of both arterial hypertension (51% in males vs. 18% in females, *p* < 0.001) and obstructive sleep apnea syndrome (34% in males vs. 3.9% in females, *p* < 0.001).

**Table 1:** Patients characteristics at baseline

Characteristic	F, *N* = 230^1^	M, *N* = 85^1^	*p*-value^2^
Age	44 (35, 54)	49 (41, 56)	0.011
BMI	36.1 (34.2, 39.4)	39.2 (36.0, 43.7)	<0.001
Excess weight	30 (25.38)	43 (34.58)	<0.001
Diabetes	5.2%	6.0%	0.8
Hyperinsulinemia	25%	35%	0.10
Hypertension	18%	51%	<0.001
OSAS	3.9%	24%	<0.001
Smoke			0.038
EX smoker	15%	28%	
No smoker	56%	45%	
Smoker	28%	28%	
Menopause	40%	NA	

^1^Median (25%, 75%)

^2^Wilcoxon rank sum test; Fisher’s exact test; Pearson’s Chi-squared test

*BMI* body mass index, *F* female, *M* male, *OSAS* obstructive sleep apnea syndrome

**Fig. 2:**
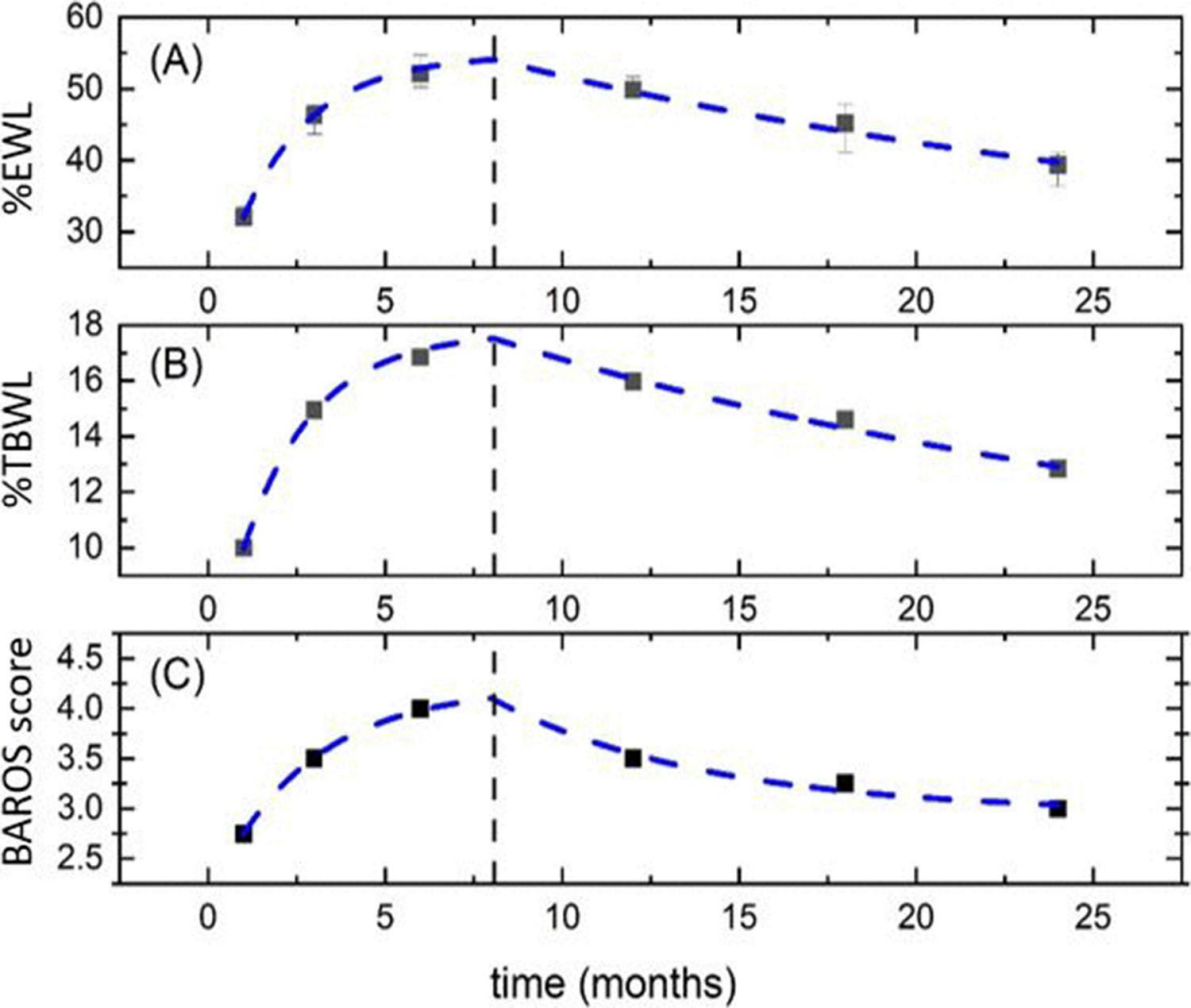
Temporal trends of %EWL, %TBWL, and BAROS score. Data are reported as median values. Equation 1 is fitted to the experimental points (blue-dashed line)

Weight loss and quality of life outcomes over 24 months of follow-up are summarized in terms of %EWL, %TBWL, and BAROS in Table 2 and Fig. 2. Notably, 73% of treated patients exhibited a %EWL >25% at 24 months, meeting the efficacy threshold for a “primary” bariatric procedure according to ASGE guidelines [5]. Similarly, 64% of subjects recorded a %TBWL >10% 2 years after ESG.

**Table 2:** Weight loss and quality of life indexes for *N* = 315 patients followed over 24 months

%EWL	Median (25%, 75%)	%TBWL	Median (25%, 75%)	BAROS score	Median (25%, 75%)
1 month	32.0 (23.5–41.3)	1 month	10.0 (8.3–12.5)	1 month	2.8 (2–3.5)
3 months	46.3 (34.9–56.9)	3 months	14.9 (11.8–18.1)	3 months	3.5 (2.8–4.8)
6 months	52.2 (37.0–68.1)	6 months	16.9 (12.9–21.4)	6 months	4 (3–5)
12 months	49.9 (30.4–66.5)	12 months	16.0 (10.4–21.7)	12 months	3.5 (2.5–5)
18 months	45.2 (23.0–63.9)	18 months	14.6 (6.9–22.2)	18 months	3.3 (1.5–5)
24 months	39.3 (17.6–58.4)	24 months	12.8 (6.41–19.4)	24 months	3 (1.5–4.6)

*%EWL* percentage of excess weight loss, *%TBWL* percentage of total body weight loss

Since the experimental data showed significant deviations from normality, the trends are presented in terms of the median and corresponding confidence interval. This trend is consistent with the model described in equation 1, which consists of an exponential increase up to time *tc*, followed by an exponential decrease. To reduce the number of fitting parameters, we assumed an equal value of *tc* for all three curves, supported by a visual examination of the experimental trends in Fig. 2. For all three parameters, the joint model suggests a critical time *tc* of about 8.3±0.9 months. Before this time point, there is a rapid weight loss (%EWL and %TBWL) and improvement in the BAROS score. We also observed that %EWL and %TBWL had a shorter and comparable time constant *tG* (growth time) of about 2.0±0.6 months, while the BAROS score showed a longer *tG* of 2.7±1.3 months.

**Fig. 3:**
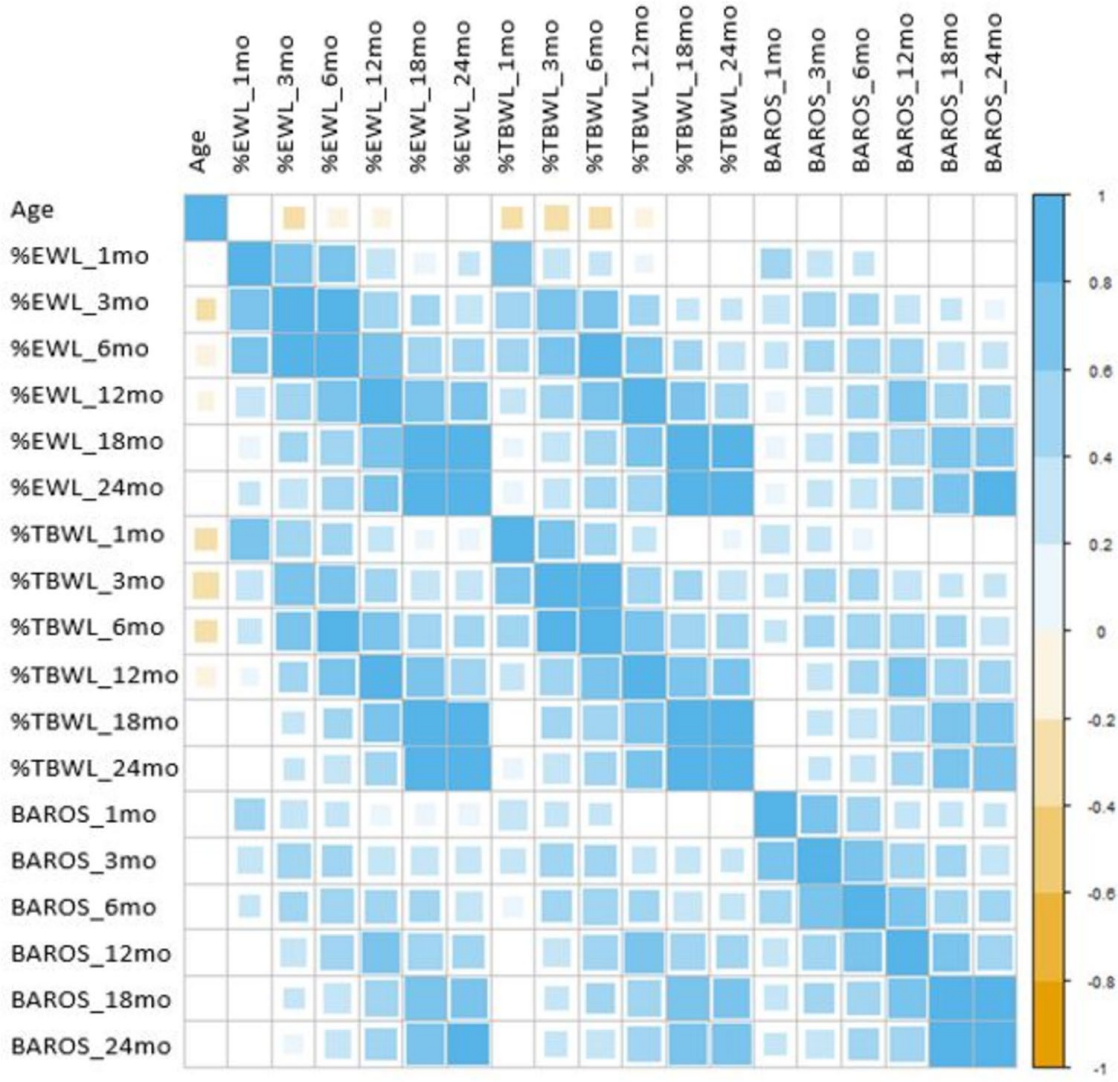
Correlation matrix among age, %EWL %TBWL, and BAROS score at different time points. Data are presented using Spearman’s correlation coefficient. White cells indicate non-significant correlations. A double scale is used to indicate the strength of the correlation. Larger and more intense squares indicate stronger positive (blue) and negative (gold) correlations

The close correlation between weight loss and quality of life indexes is further explored in Fig. 3, where we present a correlation matrix among age, %EWL, %TBWL, and BAROS, measured at different time points.

Overall, %EWL and %TBWL exhibit a more pronounced cross-correlation compared to the BAROS index. Age displays a significant negative correlation with weight loss during the early follow-up stages, which decreases over time, becoming insignificant at 24 months.

To investigate the potential presence of predictors for success or failure, we divided our population into two categories, namely “good responders” and “poor responders.” As most of the treated patients exceeded the threshold of 25% of %EWL at 24 months, we extracted the median %EWL value of approximately 39.4% at 24 months which was chosen as the threshold to differentiate between the two groups. Selecting the estimated median %EWL value at 24 months as the success definition threshold provided greater statistical power, resulting from a better balance between the two groups. In Table 3, we present an analysis of baseline parameters and comorbidities in the two groups at 24 months. Interestingly, the %EWL at 1 month after ESG was significantly higher in “good responders” compared to “poor responders” (*p* = 0.001).

**Table 3:** Baseline parameters, comorbidities, and %EWL at 1 month in “poor responders” and “good responders”

Characteristic	Good responders, *N* = 106^1^	Poor responders, *N* = 45^1^	*p*-value^2^
Gender			0.080
F	63%	78%	
M	37%	22%	
Age	48 (37.55)	48 (32.56)	0.7
Excess weight	34 (28.47)	38 (28.45)	0.6
Diabetes	7.5%	6.7%	>0.9
Hyperinsulinemia	25%	33%	0.3
Hypertension	33%	42%	0.3
OSAS	9.4%	13%	0.6
Smoke			0.4
EX smoker	17%	23%	
No smoker	51%	56%	
Smoker	31%	21%	
Menopause	42%	37%	0.6
EWL%_1	33% (25–41%)	26% (22–36%)	0.040
BMI class			0.7
30–34.9	20%	16%	
35–39.9	51%	49%	
>40	29%	36%	

^1^%; median (25%, 75%)

^2^Pearson’s Chi-squared test; Wilcoxon rank sum test; Fisher’s exact test

*BMI* body mass index, *F* female, *M* male, *OSAS* obstructive sleep apnea syndrome

**Fig. 4:**
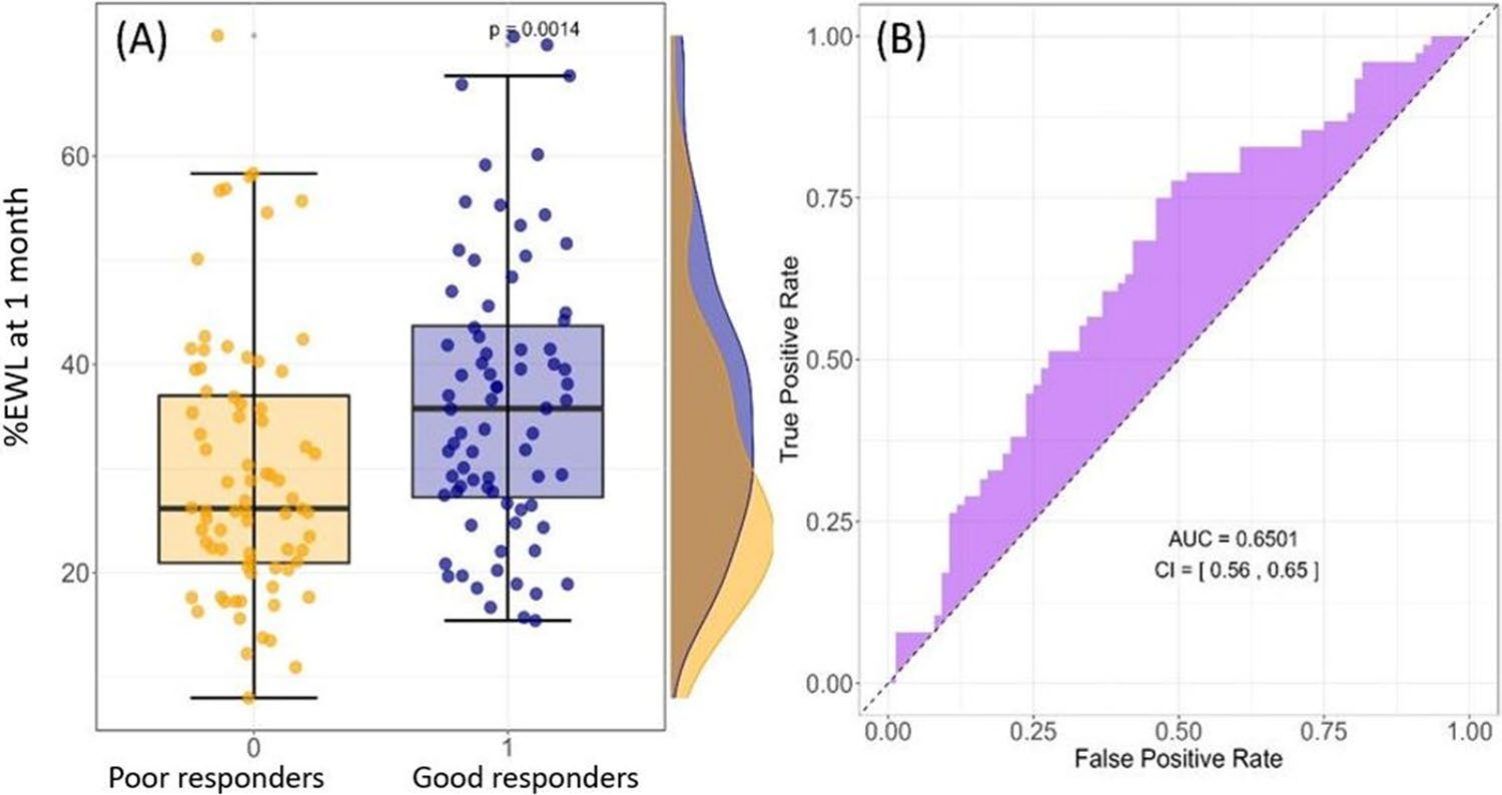
**A** Box plot analysis of %EWL at 1 month in “poor responders” (EWL%<39% at 24 months) and “good responders” (defined according to EWL%≥39% at 24 months), which are indicated with 0 and 1, respectively. **B** ROC curve analysis for evaluating the performance of %EWL in distinguishing between the two groups

Figure 4A displays a box plot analysis of %EWL data in “poor responders” and “good responders.” Consistent with prior findings, “good responders” exhibited a median %EWL value 10% higher than “poor responders” at 1 month after ESG.

Figure 4B showcases an analysis using ROC curves of the %EWL parameter measured at the 1-month follow-up, aiming to discern performance between the two groups. The curve demonstrates an AUC (area under the curve) value of 65%, significantly different from the value of 0.5 achieved by a random classifier. This supports the hypothesis that the measured parameter can be used for developing an outcome predictor.

Based on the information depicted in Fig. 4, we estimated a threshold for the excess weight loss percentage (%EWL) to effectively discriminate between “good responders” and “poor responders” at 1 month by employing Youden’s index method, a widely used technique for identifying an optimal threshold in binary classification scenarios. Through this approach, we ascertained an approximate %EWL of 31.7% as an “early threshold for success,” while considering both sensitivity and specificity.

## Discussion

In the past 10 years, endoscopic sleeve gastroplasty emerged as an effective and minimally invasive bariatric treatment enriching the available tools to fight the obesity pandemic. Several studies reported the safety and efficacy of ESG, including a recent randomized controlled trial showing an additional 12.6% of %TBWL in patients undergoing ESG compared with participants undergoing moderate-intensity lifestyle modifications alone [6, 8–14]. Our study further confirms the efficacy of ESG up to 2 years after the procedure, with 73% and 64% of treated subjects showing a %EWL >25% and a %TBWL >10%, respectively, which is consistent with the data reported in the ESG group of MERIT trial [14]. Furthermore, we observed no severe adverse events on a pretty large cohort of patients thus supporting the favorable ESG safety data [14, 21].

Our model allows a visual representation of the weight loss and quality of life outcomes over time. As anticipated, the temporal trends of the three parameters are closely interlinked. Our data show a rapid weight loss and improvement in quality of life reaching a peak at about 8–9 months after the procedure, followed by a slow decrease. As such, the time point at which we expect to see the best results is about 9 months after the procedure. However, the following decrease is slow and exponential towards a plateau suggesting that a large part of the results achieved are maintained over time.

Although the values are consistent within the experimental uncertainty, the higher tG of the BAROS score suggests that the increase in this index is delayed of about 1 month compared to %EWL and %TBWL, while the decrease in BAROS seems to slightly precede that of the weight parameters. As the BAROS score includes both %EWL and parameters of patients’ well-being, this evidence may be probably due to the patient’s need to personally experience physical improvement before achieving greater psychological well-being and awareness of quality-of-life improvement. Further, the BAROS score seems to decrease before weight loss parameters. In this regard, we hypothesize that patients may experience psychological discomfort caused by the saturation phase of physical improvements in terms of weight. However, the data presented do not allow us to verify this hypothesis. Some studies have already described the relationship between endoscopic bariatric treatments and improvement in quality of life. A metanalysis by Gadd et al. including studies on several bariatric endoscopic techniques showed that they may improve short-term quality of life and mental health alongside weight loss [22]. With a specific view on ESG, Mehta et al. showed a continuous positive association between the maximum weight loss achieved after ESG and the improvement in quality of life assessed with several validated questionnaires [23]. Further, Fiorillo et al. evaluated the Gastrointestinal Quality of Life index in a matched cohort study comparing ESG and laparoscopic sleeve gastrectomy (LSG), showing that despite the inferior weight loss (%EWL 39.9% ESG vs. 54.9% LSG, *p* = 0.01), ESG was associated with better quality of life compared with LSG, with definite advantage for the gastrointestinal symptoms [24]. In this regard, several observational studies and metanalysis comparing ESG and surgical restrictive interventions, mainly LSG, confirmed that restrictive surgery is superior to ESG in terms of weight loss, though ESG showed a better safety profile [25–28]. A such, the lower invasiveness and the favorable safety profile are strong points of the ESG, making it more acceptable than its surgical counterpart.

As obesity is a complex multifactorial disease, the results of bariatric treatments, including gastric suturing, may vary considerably between patients. As such, the identification of success predictors may have a relevant impact on clinical practice. Our analysis failed to find baseline characteristics that may predict success.

First, the patient’s gender does not seem to impact ESG outcomes, despite significantly higher age, BMI, and prevalence of comorbidities in the male cohort, as typically observed in bariatric cohorts [29]. The presence of comorbidities such as diabetes, hyperinsulinemia, hypertension, or OSAS were similar between the good and poor responders. Similarly, smoking habits and menopause in women do not appear to be indicative of potential predictive value. This is not bad news as both these conditions have been related to weight changes in the general populations, while seem to not affect outcomes of ESG. Previous studies reported that younger age is a predictor for better weight loss, probably because young people are more able to change their dietary and behavioral habits [9, 14, 15]. However, our analysis suggests that the initial stages witness greater weight loss among the younger population, while a largely similar trend emerges between younger and older patients in the long run. This evidence confirms what was reported in a previous study, supporting the use of ESG as a valid therapeutic strategy also in elder patients with obesity [30].

Although our analysis did not allow us to find a basal predictor, we observed a statistically significant difference in terms of %EWL between good and poor responders at 1 month (33% (25–41%) vs. 26% (22–36%), *p* = 0.040). Further, as no cases of stenosis or persistent vomiting occurred after ESG, the impact of these events on weight loss can be ruled out. Hence, the %EWL at 1 month may serve as a very early predictor of success at 24 months after ESG, with acceptable reliability (Fig. 4). The absolute difference in terms of %EWL between the two groups present a small overall effect size, consistent with the onset of a weight loss process that will then unfold over several months. This initial small effect size corresponds to a wide interquartile range associated with the substantial inter-individual variability among different subjects, a bottleneck that our study shares with similar investigations. Nevertheless, the substantial sample size in our study allows us to achieve statistically significant differences, both in Fig. 4 and in the AUC value. Furthermore, the statistically significant correlation shown between weight loss in the first month and the long-term value is notable. This data confirms that reported by Sharaiha et al. [13]. However, our analysis is reinforced by setting the success threshold above 25% to achieve more statistical power and allowed us to estimate a novel threshold of 1 month %EWL to detect patients at “risk of failure” in the long term, which is particularly relevant to optimize post-operative care. According to our estimation, patients not reaching the %EWL threshold of 31.7% at 1 month after ESG may be identified as at risk of poor results in the long term. For instance, these patients may benefit from additional treatments such as pharmacological drugs, including GLP-1 analogs, psychological support, or even repeating ESG [31, 32].

This study is limited by the retrospective design, the lack of a control group, and the single-center nature limiting generalization of data and intrinsically characterized by a potential selection bias. Further, our analysis is not extended beyond 2 years and would need long-term confirmation (at least 3 and 5 years) when a proportion of patients may experience weight regain due to the chronic relapsing nature of obesity. Despite the pretty long period of recruitment, only a limited percentage of patients reached long-term follow-up, preventing us from having sufficient statistical power to perform a proper analysis beyond 2 years of follow-up. Indeed, loss of follow-up is a common and big issue in routine clinical practice, especially in patients with obesity, and this is difficult to overcome. Despite of that, the analysis was conducted on a pretty large number of patients for a monocentric study and allowed us to find a simple and easy-to-use parameter such as the %EWL at 1 month to modulate the post-operative management from an early stage. Certainly, our findings should be verified and validated in future ad hoc designed prospective multicenter studies. Further, with a view to a personalized medicine, other potential factors that may predict ESG outcomes should be investigated, such as the hormonal profiles and the intestinal microbiome, whose modulation could be integrated the endoscopic treatment of obesity.

## Conclusions

Our study confirms that ESG is a safe and effective bariatric treatment that can be offered to a wide range of patients. According to our analysis, no baseline parameters, including age, gender, comorbidities, smoking habits, and menopause, showed potential predictive value of success of ESG. However, weight loss at 1 month seems to significantly predict ESG results at 2 years and should be taken into account for early identification of patients at risk of poor results in the long term who may benefit from additional treatments.

## References

[1] World Health Organization. Obesity and overweight. Available online: https://www.who.int/news-room/fact-sheets/detail/obesity-and-overweight. Accessed 30 Dec 2023.

[2] Acosta A, Streett S, Kroh MD (2017). White paper AGA: POWER - practice guide on obesity and weight management, education, and resources. Clin Gastroenterol Hepatol Off Clin Pract J Am Gastroenterol Assoc.

[3] Sullivan S, Kumar N, ASGE Bariatric Endoscopy Task Force (2015). ASGE position statement on endoscopic bariatric therapies in clinical practice. Gastrointest Endosc.

[4] Wharton S, Serodio KJ, Kuk JL (2016). Interest, views and perceived barriers to bariatric surgery in patients with morbid obesity: bariatric surgery interest & barriers. Clin Obes.

[5] Ginsberg GG, Chand B, Cote GA (2011). A pathway to endoscopic bariatric therapies. Gastrointest Endosc.

[6] Abu Dayyeh BK, Rajan E, Gostout CJ (2013). Endoscopic sleeve gastroplasty: a potential endoscopic alternative to surgical sleeve gastrectomy for treatment of obesity. Gastrointest Endosc.

[7] Abu Dayyeh BK, Acosta A, Camilleri M (2017). Endoscopic sleeve gastroplasty alters gastric physiology and induces loss of body weight in obese individuals. Clin Gastroenterol Hepatol Off Clin Pract J Am Gastroenterol Assoc.

[8] Lopez-Nava G, Galvão MP, Bautista-Castaño I (2017). Endoscopic sleeve gastroplasty for obesity treatment: two years of experience. ABCD Arq Bras Cir Dig São Paulo.

[9] Sharaiha RZ, Kumta NA, Saumoy M (2017). Endoscopic sleeve gastroplasty significantly reduces body mass index and metabolic complications in obese patients. Clin Gastroenterol Hepatol Off Clin Pract J Am Gastroenterol Assoc.

[10] Lopez-Nava G, Sharaiha RZ, Vargas EJ (2017). Endoscopic sleeve gastroplasty for obesity: a multicenter study of 248 patients with 24 months follow-up. Obes Surg.

[11] Graus Morales J, Crespo Pérez L, Marques A (2018). Modified endoscopic gastroplasty for the treatment of obesity. Surg Endosc.

[12] Alqahtani A, Al-Darwish A, Mahmoud AE (2019). Short-term outcomes of endoscopic sleeve gastroplasty in 1000 consecutive patients. Gastrointest Endosc.

[13] Sharaiha RZ, Hajifathalian K, Kumar R (2021). Five-year outcomes of endoscopic sleeve gastroplasty for the treatment of obesity. Clin Gastroenterol Hepatol.

[14] Abu Dayyeh BK, Bazerbachi F, Vargas EJ (2022). Endoscopic sleeve gastroplasty for treatment of class 1 and 2 obesity (MERIT): a prospective, multicentre, randomised trial. Lancet Lond Engl.

[15] Barrichello S, Hourneaux De Moura DT, Hourneaux De Moura EG (2019). Endoscopic sleeve gastroplasty in the management of overweight and obesity: an international multicenter study. Gastrointest Endosc.

[16] Dal Prà C, Fabris R. Obesity and gender differences. Ital J Gend-Specif Med. 2020; 10.1723/3297.32669.

[17] Oria HE, Moorehead MK (1998). Bariatric analysis and reporting outcome system (BAROS). Obes Surg.

[18] Brethauer SA, Kim J, El Chaar M (2015). Standardized outcomes reporting in metabolic and bariatric surgery. Surg Obes Relat Dis.

[19] Wei T, Simko V, Levy M (2017). Package “corrplot”. Statistician.

[20] Napodano C, Callà C, Fiorita A (2021). Salivary biomarkers in COVID-19 patients: towards a wide-scale test for monitoring disease activity. J Pers Med.

[21] Storm AC, Abu Dayyeh BK (2019). Endoscopic sleeve gastroplasty for obesity: defining the risk and reward after more than 1600 procedures. Gastrointest Endosc.

[22] Gadd N, McIntosh A, Fear-Keen B (2020). Do endoscopic bariatric procedures improve postprocedural quality of life and mental health? A systematic review and meta-analysis. Obes Surg.

[23] Mehta A, Hajifathalian K, Shah SL (2022). Quality of life, mental health, and weight loss outcomes following endoscopic sleeve gastroplasty. J Gastrointest Surg.

[24] Fiorillo C, Quero G, Vix M (2020). 6-Month gastrointestinal quality of life (QoL) results after endoscopic sleeve gastroplasty and laparoscopic sleeve gastrectomy: a propensity score analysis. Obes Surg.

[25] Novikov AA, Afaneh C, Saumoy M (2018). Endoscopic sleeve gastroplasty, laparoscopic sleeve gastrectomy, and laparoscopic band for weight loss: how do they compare?. J Gastrointest Surg Off J Soc Surg Aliment Tract.

[26] Lopez-Nava G, Asokkumar R, Bautista-Castaño I (2021). Endoscopic sleeve gastroplasty, laparoscopic sleeve gastrectomy, and laparoscopic greater curve plication: do they differ at 2 years?. Endoscopy..

[27] Fayad L, Adam A, Schweitzer M (2019). Endoscopic sleeve gastroplasty versus laparoscopic sleeve gastrectomy: a case-matched study. Gastrointest Endosc.

[28] Marincola G, Gallo C, Hassan C (2021). Laparoscopic sleeve gastrectomy versus endoscopic sleeve gastroplasty: a systematic review and meta-analysis. Endosc Int Open.

[29] Van Olst N, Reiber BMM, Vink MRA, et al. Are male patients undergoing bariatric surgery less healthy than female patients? Surg Obes Relat Dis. Published online February 2023: S1550728923000898. 10.1016/j.soard.2023.02.015 10.1016/j.soard.2023.02.015 36967264

[30] Matteo MV, Bove V, Pontecorvi V (2022). Outcomes of endoscopic sleeve gastroplasty in the elder population. Obes Surg.

[31] Badurdeen D, Hoff AC, Hedjoudje A (2021). Endoscopic sleeve gastroplasty plus liraglutide versus endoscopic sleeve gastroplasty alone for weight loss. Gastrointest Endosc.

[32] Lopez-Nava G, Asokkumar R, Negi A (2021). Re-suturing after primary endoscopic sleeve gastroplasty (ESG) for obesity. Surg Endosc.

